# Patterns of variation in diversity of the Mississippi river microbiome over 1,300 kilometers

**DOI:** 10.1371/journal.pone.0174890

**Published:** 2017-03-28

**Authors:** Jason T. Payne, Justin J. Millar, Colin R. Jackson, Clifford A. Ochs

**Affiliations:** Department of Biology, University of Mississippi, University, Mississippi, United States of America; Loyola University Chicago, UNITED STATES

## Abstract

We examined the downriver patterns of variation in taxonomic diversity of the Mississippi River bacterioplankton microbiome along 1,300 river kilometers, or approximately one third the total length of the river. The study section included portions of the Upper, Middle, and Lower Mississippi River, confluences with five tributaries draining distinct sub-basins, river cities, and extended stretches without major inputs to the Mississippi. The composition and proportional abundance of dominant bacterial phyla was distinct for free-living and particle-associated cells, and constant along the entire reach, except for a substantial but transient disturbance near the city of Memphis, Tennessee. At a finer scale of taxonomic resolution (operational taxonomic units, OTUs), however, there were notable patterns in downriver variation in bacterial community alpha diversity (richness within a site) and beta diversity (variation in composition among sites). There was a strong and steady increase downriver in alpha diversity of OTUs on suspended particles, suggesting an increase in particle niche heterogeneity, and/or particle colonization. Relatively large shifts in beta diversity of free-living and particle-associated communities occurred following major tributary confluences and transiently at Memphis, while in long stretches between these points diversity typically varied more gradually. We conclude that the Mississippi River possesses a bacterioplankton microbiome distinct in diversity from other large river microbiomes in the Mississippi River Basin, that at major river confluences or urban point sources its OTU diversity may shift abruptly and substantially, presumably by immigration of distinct external microbiomes, but that where environmental conditions are more stable along the downriver gradient, microbiome diversity tends to vary gradually, presumably by a process of successional change in community composition.

## Introduction

All ecosystems have a microbial community, or microbiome, from the tissues and organs of individual animal and plant hosts [1, 2], to aquatic or terrestrial environments [3]. The microbiome is involved with essential ecosystem processes whether it is contributing to host metabolism [4–6] or biogeochemical cycling of nutrients at regional or global spatial scales [7]. Hence, variation in microbiome composition may have implications for the functional attributes, services, or adaptability of the ecosystem [8].

A fluid ecosystem may exhibit change in microbiome composition from one region to another along the path of flow. Examples of such systems include the human gastrointestinal tract [9] and lotic systems such as streams or rivers [10, 11]. Variation in diversity of the microbiome along the flow path may be slow and gradual along regions of weak gradients of environmental change and selection, or occur abruptly at nexuses with other ecosystems [11–13]. Examination of microbiome diversity along a fluid ecosystem having both major confluences, and long stretches of slow, gradual change between these interfaces, may reveal the relative importance of abrupt supplementation of distinct, external microbiomes, versus adaptation and gradual selection moving downriver.

The main objective of this study was to determine the spatiotemporal pattern of variation in planktonic microbial community diversity of the Mississippi River as it flows downriver over 1,300 river kilometers (rkm), converging intermittently with other rivers that drain large sub-basins of the Mississippi River Basin. We hypothesized that changes in the diversity of the microbiome would be relatively large and abrupt following mixing at confluences, but more gradual with increasing downriver distance from confluences. This hypothesis was predicated on a prior finding that each of these rivers has a distinct microbiome [14]; the repeatability of this finding and its implications were tested independently in this study. By following microbiome diversity with river flow from directly above, to close below, to far between, and within tributary inputs we were able to evaluate the likelihood of these two proposed mechanisms, sudden tributary inoculation vs. gradual environmental selection [11, 15, 16], on the pattern of downriver variation in compositional diversity of the Mississippi River microbiome.

For analysis of the microbiomes of the Mississippi River and its tributaries, we utilized barcoded next generation Illumina sequencing of the bacterial 16S rRNA gene [17]. This approach facilitates detailed characterization of microbiome composition along temporal and spatial gradients. When viewed at a low level of taxonomic resolution a microbiome may appear fairly homogenous among ecosystems having broadly shared environmental characteristics [18–21]. However, when viewed at a high level of taxonomic resolution, shifts in microbiome composition may become evident with consequences for microbiome function [22]. Here we compare downriver spatial variation in the microbiome(s) at a low level of taxonomic resolution, phylum-level classification of bacterial OTUs (operational taxonomic units); and at a high level of resolution, individual bacterial OTUs.

## Materials and methods

### The Mississippi River system

The Mississippi River Basin encompasses 41% of the lower United States and is composed of six major sub-basins, including the Upper Mississippi River Basin, the Missouri River Basin, the Ohio/Tennessee River Basin, the Arkansas/White/Red River Basin, and the Lower Mississippi River Basin. Each sub-basin is distinct in land-use practices, physiography, climate, and the physicochemical properties of its major rivers [23, 24]. The 3,700 rkm Mississippi River itself can be divided into three sections based on where it merges with its two largest tributaries The Upper Mississippi River (UMR) has its headwaters at Lake Itasca, Minnesota, and ends at the confluence with the Missouri River, at St. Louis, Missouri. The latter 1,059 rkm of the UMR section consists of a series of large pools created by navigation dams. The Middle Mississippi River (MMR) is unimpounded and free-flowing and extends about 310 rkm from the Missouri River confluence to the confluence with the Ohio River, at Cairo, Illinois. The Lower Mississippi River (LMR) is also free-flowing and extends 1,600 rkm from the Ohio River confluence to its outlet into the Gulf of Mexico.

### Sampling the Mississippi River network

**Fig 1:**
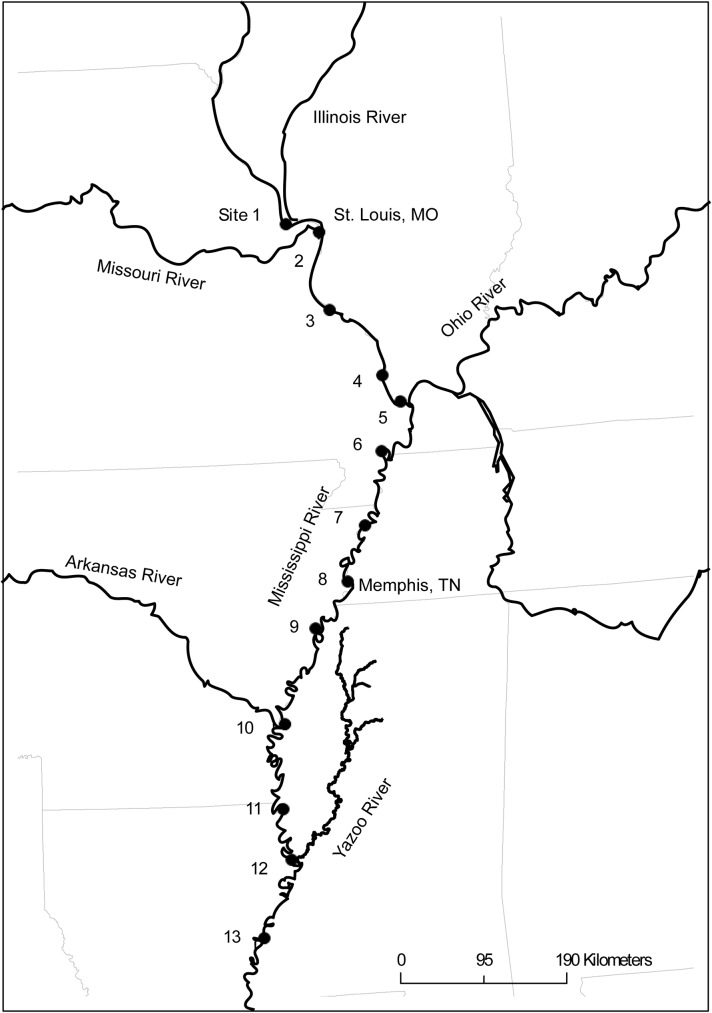
Sample sites on the Mississippi River. Sample sites are indicated by closed circles and referred to by number, from site 1 to site 13, in order from north to south (Table 1).

Water samples were collected in sequence from a series of thirteen sample sites on the Mississippi River extending from just above St. Louis, Missouri to Natchez, Mississippi, a distance of 1,300 rkm. Additionally, samples were collected from the mouths of five tributaries to the Mississippi, each of which drains an important sub-basin. Only one site per day was sampled on the Mississippi starting on July 22, 2013 at the north-most site (site 1) and ending on August 03, 2013 (site 13) (Table 1, Fig 1). Sample site 1 was located in pool 26 of the UMR. All other sample sites were in unimpounded, free-flowing portions of the Mississippi or the tributaries.

**Table 1:** Sites sampled on the Mississippi River and its major tributaries, July-August 2013.

		Mississippi River				
Date	River^a^	Site^b^	rkm^c^	Location	Discharge (m/s)^d^	Depth (m)
7/22	UMR	1	1,889	N 38 56.788, W 90 28.497	1,880	3.4
7/22	Illinois			N 38 57.897, W 90 29.778	435	6.7
7/23	UMR	2	1,851	N 38 49.688, W 90 06.685	2,315	7.6
7/23	Missouri			N 38 49.587, W 90 08.054	1,479	4.8
7/24	MMR	3	1,733	N 38 00.196, W 90 02.823	3,794	9.1
7/25	MMR	4	1,617	N 37 20.077, W 89 28.985	6,098	8.8
7/26	MMR	5	1,537	N 37 01.596, W 89 12.993	5,737	13.5
7/26	Ohio			N 37 01.542, W 89 10.531	7,584	12.1
7/27	LMR	6	1,432	N 36 34.716, W 89 31.331	13,321	10.1
7/28	LMR	7	1,304	N 35 53.143, W 89 46.106	12,856	15.4
7/29	LMR	8	1,191	N 35 13.442, W 90 04.502	13,960	12.8
7/30	LMR	9	1,107	N 34 44.586, W 90 27.001	12,912	16.2
7/31	LMR	10	941	N 33 49.000, W 91 02.902	14,753	12.3
7/31	Arkansas			N 33 48.615, W 91 06.538	1,017	5.9
8/01	LMR	11	803	N 32 54.010, W 91 03.972	15,770	18.5
8/02	LMR	12	708	N 32 20.801, W 90 57.772	17,273	12.5
8/02	Yazoo			N 32 23.352, W 90 54.981	142	11.9
8/03	LMR	13	587	N 31 33.642, W 91 24.596	17,415	22.8

^a^UMR, Upper Mississippi River; MMR, Middle Mississippi River; LMR, Lower Mississippi River.

^b^Sample sites on the Mississippi River are numbered sequentially from north to south.

^c^rkm, river kilometers. rkm for sites on the Mississippi River starting from Head of Passes, Louisiana, which is rkm 0.

^d^Discharge data by date provided by the U.S. Geological Survey and the U.S. Army Corp of Engineers.

The daily sampling regime was designed to roughly keep up with river flow velocity, estimated during our sample period in our sampling reach at 100–150 km per day. This estimate is based on continuous measurements made by the United States Geological Survey at Belle Chasse, Louisiana, (https://waterdata.usgs.gov/nwis/uv?site_no=07374525), adjusted for a river velocity of about 1.6 km/h faster above the floodgate system of the Old River Control Structure (personal communication, J. Ruskey). With the exception of the first two sample sites on the UMR, sites 1 and 2, all sample sites were separated by >80 rkm (ranging from 80 to 166 rkm; mean = 116 rkm) (Table 1).

Of the thirteen sample sites on the Mississippi, five sites were located within 3–8 rkm above confluences with the five river tributaries. Within 1 h of the time that these sites were sampled, the associated tributary was also sampled within its mouth just above the confluence. Sample sites on the Mississippi subsequent to tributary inputs were located 34 to 130 rkm below confluences to allow for full mixing of the convergent rivers.

Sampled tributaries include three that are relatively large in the percentage of water they add to the Mississippi at their confluence, and two that are relatively small in this percentage. The former tributaries are the Illinois River (contributing 19% of the summed discharge), the Missouri River (contributing 39%), and the Ohio River (contributing 57%). The two relatively small tributaries are the Arkansas River (contributing 7%), and the Yazoo River (contributing 1%) (Table 1). Henceforth, where we refer to *major* tributaries, we are referring specifically to the relatively large tributaries: the Illinois River, the Missouri River, and/or the Ohio River.

Samples were collected from 0.5 m depth at mid-river locations. River depths at sample sites (Table 1) were measured using a hand-held Hawkeye H22PX digital sonar system. All samples were collected between 1000 and 1300 h, except for on July 26 at the Middle Mississippi/Ohio confluence where collections were made between 1600–1630 h. Each collection consisted of three sterilized 1-L Nalgene bottles for determination of water chemical, physical, and biological parameters, and three sterilized 500-mL Nalgene bottles for determination of bacterial community structure. Each sample bottle was rinsed three times with river water prior to being filled with sample. Samples were stored in river water in insulated coolers to maintain ambient temperature during transportation (1–2 h) before being processed. All samples used in this study were collected from public river waterways for which permission to obtain samples was not required.

### Water parameter measurements

For chemical analyses, water samples (100- or 200-mL) were filtered through ashed 47-mm diameter, 3-μm pore size Whatman GF/F filters. Filtered sample water and filters were frozen immediately for subsequent chemical analyses. Orthophosphate, nitrate, and ammonium, were measured from filtered water following standard colorimetric methods [25], and total dissolved nitrogen and total dissolved organic carbon were measured using a Shimadzu TOC-L Total Organic Carbon analyzer. Particulate carbon and nitrogen were measured from filters concurrently by dynamic combustion using a Perkin Elmer 2400 Series II CHNS/O analyzer, and total suspended solids concentration was measured by gravimetry. Chlorophyll *a* was extracted from filters in 90% NH_4_OH-buffered acetone for 24 h at 5°C and measured by spectrophotometry [25]. Water temperature and pH were measured on-site using a YSI Professional Plus multiparameter meter.

### DNA extraction and sequencing

For analysis of bacterial community composition, the 500-ml sample bottles were mixed, and sub-samples (100 mL per bottle) withdrawn. These sub-samples were filtered in series through a sterile Millipore 3-μm pore-size polycarbonate filter to collect particle-associated or relatively large bacteria, and a sterile Millipore 0.22-μm pore-size polyethersulfone filter to collect free-living bacteria [11, 14]. Each filtration was performed at <5 mm Hg and filters were kept at -20°C until DNA extraction.

PowerWater DNA isolation kits (MoBio, Carlsbad, California) were used to extract DNA from each filter. The V4 region of 16S rRNA genes was amplified using forward (5’-GTGCCAGCMGCCGCGGTAA) and reverse (5’-GGACTACHVGGGTWTCTAAT) primers coupled with dual index barcodes that are optimized for Illumina MiSeq sequencing [17]. Amplifications were run under conditions defined previously [26]. Briefly, DNA was initially denatured at 95°C for 2 min, then run through 30 cycles of denaturation (95°C for 20 s), annealing (55°C for 15 s), and elongation (72°C for 2 min), and ended with a final elongation step (72°C for 10 min). Amplified DNA was normalized by sample using SequalPrep Normalization Plates (Life Technologies, Grand Island, New York), pooled, and sequenced by the Molecular and Genomics Core Facility at the University of Mississippi Medical Center using an Illumina MiSeq. Sequence data were processed using mothur following guidelines set forth by Schloss *et al*. [27] and Kozich *et al*. [17].

Sequences were aligned to reference V4 sequences using the SILVA rRNA database [28] and sequences that did not align with the V4 region and homopolymers >8 bp were discarded. Prior to classification, sequences nearly identical (<2 bp differences) to one another were merged [26], and potential chimeras identified using UCHIME [29] and removed. Taxonomic classification of sequences was performed using the Greengenes database [30], a database more suitable for classifying 16S rRNA sequences than the SILVA database used to align sequences [31]. Any sequence classified as being non-bacterial in origin (e.g. sequences derived from chloroplasts, mitochondria, Archaea, Eukarya, or unknown sources) was removed. All bacterial sequence reads were clustered into OTUs based on ≥97% similarity and identified to the highest possible taxonomic resolution. All sequences were deposited in the NCBI SRA database under the BioProject ID PRJNA356973.

### Sequence analyses

Sequence data were visualized and analyzed using the phyloseq version 1.14.0 [32], vegan version 2.4–1 [33], and ggplot2 version 2.1.0 [34] packages implemented in R version 3.2.4 revised [35]. Two free-living bacterial samples obtained from the Mississippi River were removed from the data set because of poor sequencing depth (<5,000 reads per sample) [36].

Patterns at the phylum-level in downriver community composition were examined using average relative abundances of OTU-reads grouped by phylum or “unclassified” for OTUs classified to domain Bacteria only. Average relative abundances were calculated from replicate samples for each site on the Mississippi. Singleton and other rare OTUs represented by reads that comprised <0.001% of total reads were removed to reduce inclusion of potentially erroneous sequence data into analyses.

For analysis of beta diversity, sample sequences were normalized to provide the same number of reads per sample. Before normalization, rare OTUs, defined as above, were removed. Then, each sample of bacterial sequences was randomly subsampled to equal sequencing depths of samples having the fewest number of reads. From this process we obtained for all rivers, 6,603 and 9,617 reads per sample, for free-living and particle-associated bacteria, respectively. For the Mississippi River alone, we obtained 6,587 and 11,581 reads per sample, for free-living and particle-associated bacteria, respectively. To quantify beta diversity, we used the Bray-Curtis dissimilarity index that compares proportional abundances of OTUs among samples. To visualize patterns in beta diversity, dissimilarity matrices were ordinated by non-metric multidimensional scaling (NMDS). Dissimilarity matrices were analyzed statistically by permutational multivariate analysis of variation (*adonis*, permutations = 999) to test the null hypothesis of no differences in centroids of communities grouped by river or, for the Mississippi alone, the river section [37]. Removal of rare OTUs, sequence-number normalizations, dissimilarity calculations, and ordinations were performed using the functions prune_taxa, rarefy_even_depth, phyloseq::distance, and ordinate in the phyloseq package, respectively. All random number generator seeds were set to “1000” for standardizations and statistical analyses to ensure that these analytical results were reproducible [32].

To determine downriver patterns in microbiome alpha diversity, the richness of OTUs obtained from untrimmed sequence data (e.g. including singleton and rare OTUs) was analyzed using the estimate_richness function in the phyloseq package [32]. Samples were randomly subsampled (100 iterations) to equal sequencing depths of samples having the fewest number of sequence reads. Free-living and particle-associated bacterial samples were normalized to 6,690 reads per sample, the fewest number of reads among all samples. After subsampling, the number of OTUs was averaged across replicate samples for each of the sites on the Mississippi River and linear regression used to determine the relationships of OTU richness and downriver distance.

## Results

### Water parameters

There were distinct differences in chemical, physical, and biological parameters among the three sections of the Mississippi River and its tributaries (Table 2). Here, we focus on inputs, and corresponding effects, of the three major tributaries (Illinois, Missouri, Ohio Rivers) to the Mississippi, and on downriver patterns in the Mississippi. Orthophosphate concentration was highest in the Illinois River, contributing to a concentration increase of 58% between adjacent sites on the UMR, before declining to 83.2 and 74.0 μg/L in the MMR and LMR, respectively. Total dissolved nitrogen, consisting mostly of nitrate, was highest in concentration in the UMR and declined with distance downriver to 3.5 and 1.9 mg/L in the MMR and LMR, respectively. Dissolved organic carbon (TOC), particulate carbon, and particulate nitrogen also declined from maximum values in the UMR to low and less variable (with the exception of TOC) values in the LMR. Total suspended solids (TSS) were high in the Missouri, contributing to a 30% increase in TSS from the UMR to the MMR. Following dilution by the Ohio River, TSS declined in the LMR to a mean of 55.6 mg/L (excluding site 9 where TSS was 276 mg/L). Chlorophyll *a* was about 20 μg/L in the UMR, increased to a mean of 26 μg/L after the confluence with the Missouri, then declined to 16.5 μg/L in the LMR. Similar spatial patterns of these parameters in the Mississippi River and its tributaries during summer have been noted in other studies as consequences of sub-basin influences [14, 23, 24, 38].

**Table 2:** Water parameter measurements from the Mississippi River and its major tributaries, July-August 2013.

Parameter^a^	UMR^b^ *n = 1*	UMR^c^ *n = 1*	MMR *n = 3*	LMR *n = 8*	Illinois *n = 1*	Missouri *n = 1*	Ohio *n = 1*	Arkansas *n = 1*	Yazoo *n = 1*
**PO**_**4**_**-P μg/L**	63.8 (11.3)	101.5 (6.4)	83.2 (6.1)	74.0 (8.8)	154.8 (70.1)	85.4 (8.8)	27.5 (5.2)	18.2 (0.8)	44.7 (4.2)
**NO**_**3**_**-N mg/L**	4.0 (0.1)	4.0 (0.0)	3.3 (0.1)	1.7 (0.1)	2.4 (0.0)	1.5 (0.1)	1.0 (0.0)	0.0 (0.0)	0.6 (0.0)
**NH**_**4**_**-N μg/L**	24.9 (22.2)	20.2 (1.3)	6.0 (6.6)	18.6 (17.5)	36.8 (9.0)	6.0 (10.4)	11.5 (20.0)	26.2 (2.0)	7.0 (12.1)
**TDN mg/L**	4.4 (0.1)	4.2 (0.1)	3.5 (0.2)	1.9 (0.2)	3.1 (0.0)	1.6 (0.0)	1.2 (0.0)	0.4 (0.0)	0.8 (0.0)
**TOC mg/L**	10.9 (1.0)	9.0 (1.2)	9.5 (1.3)	5.6 (1.2)	8.5 (0.6)	7.3 (0.9)		7.7 (0.2)	6.0 (0.1)
**pH**	8.2	7.7	7.8 (0.0)	7.7 (0.1)	8.1	8.0	7.4	8.0	7.3
**Part C mg/L**	3.2 (0.3)	3.1 (0.1)	3.2 (0.1)	2.2 (0.2)	2.5 (0.0)	3.0 (0.0)	1.5 (0.1)	2.6 (0.3)	3.6 (0.2)
**Part N mg/L**	0.38 (0.04)	0.40 (0.00)	0.37 (0.01)	0.23 (0.03)	0.35 (0.00)	0.35 (0.00)	0.17 (0.01)	0.36 (0.05)	0.43 (0.01)
**TSS mg/L**	69.2 (26.9)	60.2 (2.8)	78.1 (8.5)	83.2 (78.3)	36.5 (1.5)	78.7 (6.6)	39.5 (4.6)	57.5 (9.3)	121.8 (13.7)
**Temp°C**	29.5	29.2	29.4 (0.3)	28.3 (0.4)	29.0	27.5	28	28.9	30.2
**Chl *a* μg/L**	20.9 (1.4)	19.9 (1.4)	26.1 (2.8)	16.5 (1.7)	27.1 (0.9)	46.4 (1.1)	12.5 (0.5)	34.9 (2.4)	34.3 (3.9)

n, number of sites sampled per river or river section, 3 replicates per site.

TDN, total dissolved nitrogen; TOC, total dissolved organic carbon; Part C, particulate carbon; Part N, particulate nitrogen; TSS, total suspended solids; Temp, water temperature; Chl *a*, chlorophyll *a*.

^a^For the MMR and LMR, parameters are presented as means and standard deviations of site means. For the UMR and tributary rivers, parameters are presented as means and standard deviations of replicates within a site.

^b^Upper Mississippi River sampled above confluence with the Illinois River.

^c^Upper Mississippi River sampled below confluence with the Illinois River.

To compare stability of environmental conditions within river sections to between river sections, we determined the coefficient of variation (standard deviation/mean) of each parameter in Table 2 for sites within the MMR, within the LMR, and between mean values of the MMR and LMR. The UMR was excluded from this comparison because we sampled only two sites in this river section that were separated by an impoundment and a major tributary. Evident from this comparison is that for most of these parameters there was much less variation within these two river sections than between them (Table 3).

**Table 3:** Comparison of sites for variation in parameter measurements.

Parameter	Within MMR *n = 3 sites*	Within LMR *n = 8 sites*	MMR vs LMR *n = 2 sections*
**PO**_**4**_**-P**	7.4	11.9	8.3
**NO**_**3**_**-N**	4.1	8.0	45.3
**NH**_**4**_**-N**	109.3	94.1	72.4
**TDN**	5.2	8.7	41.9
**TOC**	13.9	21.4	36.5
**pH**	0.2	1.0	0.9
**Part C**	3.8	9.6	26.2
**Part N**	2.8	13.3	33.0
**TSS**	10.9	11.6	23.8
**Temp**	0.9	1.3	2.7
**Chl *a***	10.7	10.4	31.9

Parameters and parameter units as in Table 2. n, samples sizes.

Values shown are the percentage of the standard deviation/mean (coefficient of variation). For MMR vs LMR, the calculation is based on the means of the two sections.

### Sequences

After removal of potentially erroneous, chimeric, and non-bacterial reads, a total of 4,864,418 reads (length = 253 bp) of the V4 region of the 16S rRNA gene were generated. These represented a total of 28,244 bacterial OTUs in the river microbiome, from 106 samples. Particle-associated and free-living bacterial communities were investigated separately. For sample sites on the Mississippi River, 1,799,962 sequences corresponding to 8,990 OTUs (1,769,980 sequences, 2,202 OTUs following removal of singletons and other rare OTUs) were recovered from free-living samples (n = 37), and 1,934,271 sequences representing 21,149 OTUs (1,861,831 sequences, 4,889 OTUs following removal of rare OTUs) were obtained from particle-associated samples (n = 39). Hence, while the number of reads recovered from the different bacterial samples was similar, there were approximately twice as many OTUs observed in the particle-associated datasets.

An additional 567,710 sequences representing 4,366 OTUs (536,848 sequences, 523 OTUs following removal of rare OTUs) were recovered from free-living samples taken from tributaries (n = 15), while 562,475 sequences representing 10,877 OTUs (490,759 sequences, 958 OTUs following removal of rare OTUs) were obtained from particle-associated samples from tributaries (n = 15).

### Downriver patterns in bacterial phyla

**Fig 2:**
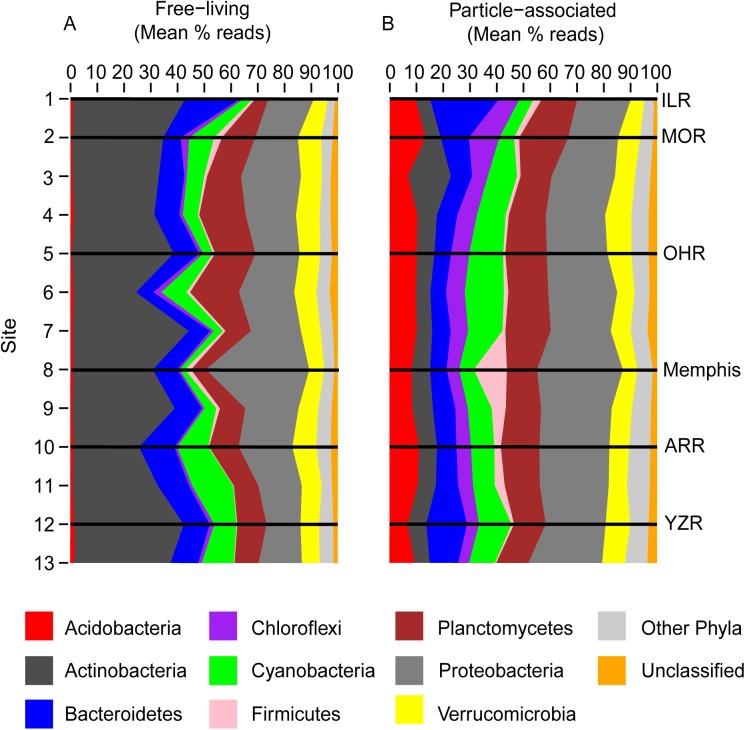
Relative abundances of dominant bacterial phyla sampled along the Mississippi River. Dominant bacterial phyla were defined as comprising >1% of all pooled reads for (A) free-living and (B) particle-associated bacterioplankton communities. Less common phyla (<1% of all pooled reads) are labeled as “Other Phyla”, and unclassified bacterial reads are labeled as “Unclassified”. Relative abundances are presented as mean percent reads, n = 2–3 per site. Sites on the Mississippi River are numbered sequentially from north to south (Table 1). Black horizontal lines indicate sites on the Mississippi River located just above confluences with tributaries (ILR = Illinois R; MOR = Missouri River; OHR = Ohio River; ARR = Arkansas River; YZR = Yazoo River) and site 8 at Memphis, Tennessee.

**Fig 3:**
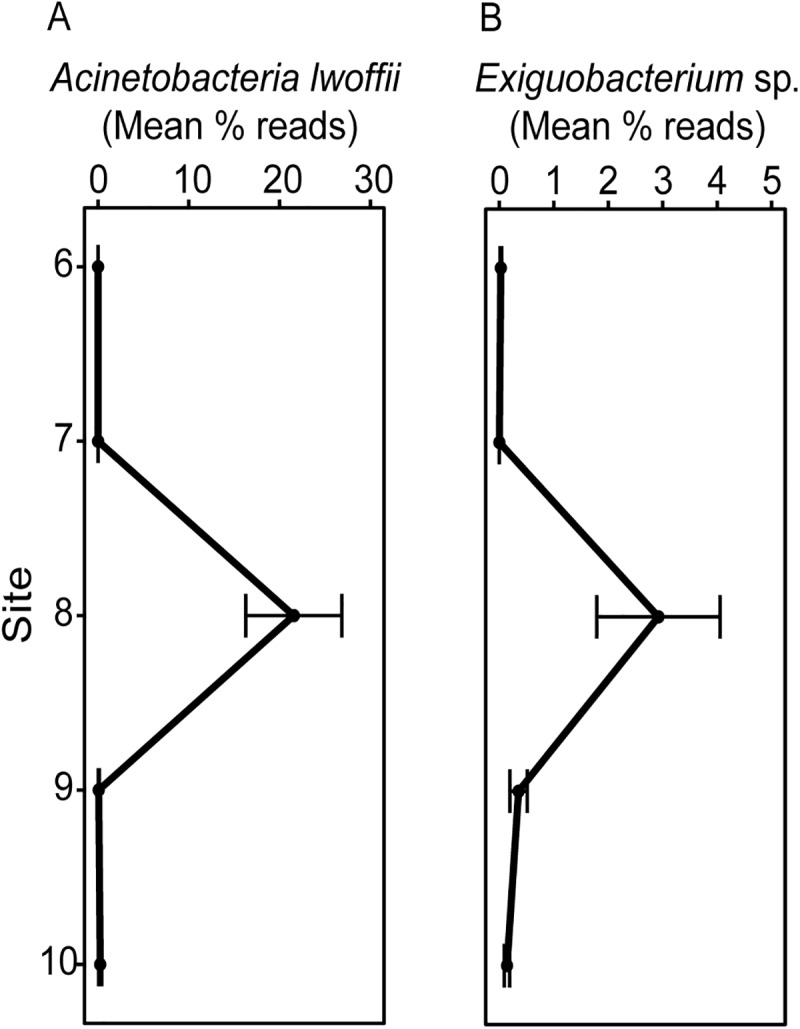
**Relative abundances of OTUs identified as (A) *Acinetobacteria lwoffii* and (B) *Exiguobacterium* sp. proximal to Memphis, Tennessee.** Relative abundances (mean percent reads ± SE, n = 3 per site) of *Acinetobacteria lwoffii* (free-living bacterioplankton) and *Exiguobacterium* sp. (particle-associated) are presented from sites sampled before, at, and after Memphis (site 8).

We identified 37 phyla of bacteria in the free-living samples taken from the Mississippi River and 49 phyla in the particle-associated fraction. “Dominant” phyla were designated as phyla consisting of >1% of all pooled reads. For both particle-associated and free-living bacteria, the same nine phyla met this criterion (Fig 2). Actinobacteria consistently accounted for the highest proportion of sequences in free-living samples along the Mississippi River, while Proteobacteria were dominant in particle-associated samples. Proportions of Bacteroidetes, Cyanobacteria, Planctomycetes, Proteobacteria, and Verrucomicrobia were similar for the two fractions. Proportions of dominant phyla generally varied only slightly between sites for both free-living and particle-associated communities. The exception was at Memphis, Tennessee, where there was a notable increase in the proportion of Proteobacteria, and a corresponding decrease in the proportion of Actinobacteria among free-living samples, and an increase in the proportion of Firmicutes (and corresponding decrease in the proportion of Cyanobacteria) in the particle-associated fraction. The increase of free-living Proteobacteria was largely attributed to increases of *Acinetobacteria lwoffii* (Fig 3A), whereas the increase of particle-associated Firmicutes at Memphis was attributed to increases of *Exiguobacterium* sp. (Fig 3B). This shift was transient, and by the following sample site 84 rkm downriver, *A*. *lwoffii* had declined to pre-Memphis proportions, while among particle-associated phyla the shift also diminished.

### Downriver patterns in alpha diversity

**Fig 4:**
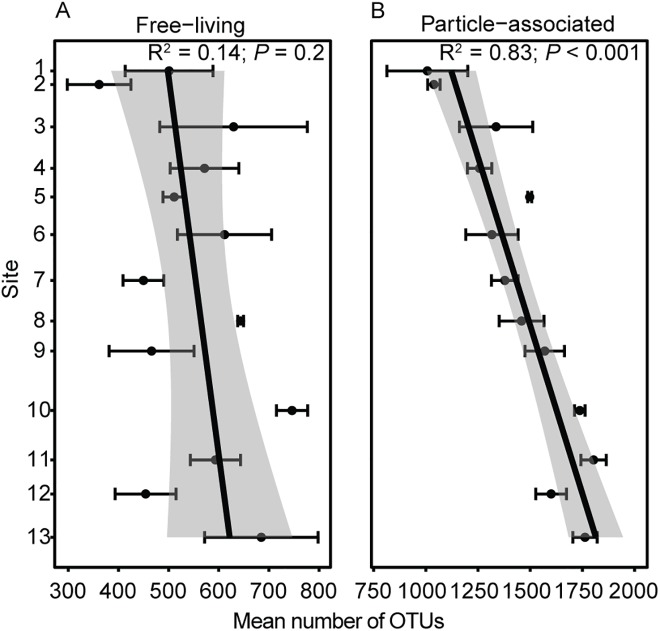
**Alpha diversity of (A) free-living and (B) particle-associated bacterioplankton communities sampled along the Mississippi River.** Alpha diversity is represented as the number of OTUs (mean ± SE) per sample site (n = 2–3). Sites on the Mississippi River are numbered sequentially from north to south (Table 1). Black lines are fitted regression lines predicting mean OTU number as a function of downriver distance. Gray auras around regression lines represent 95% confidence regions. Statistics for regressions are shown in each panel.

Alpha diversity of free-living bacteria, measured as mean OTU richness, varied from 361 ± 63 to 746 ± 31 (mean ± SE) for the Mississippi River sites, with little or no downriver pattern (Fig 4A). Particle-associated communities were more diverse, and strongly increased in richness with distance downriver, almost doubling from the north–most (1,007 ± 193 OTUs) to the south-most (1,762 ± 58 OTUs) sample site (Fig 4B). Punctuations occurred in the general trend of increasing OTU richness in particle-associated bacteria. Below the confluence with the Missouri River, richness abruptly increased by 29%, and below its next major tributary, the Ohio River, there was an abrupt decline of 12%.

### Downriver patterns in beta diversity

**Fig 5:**
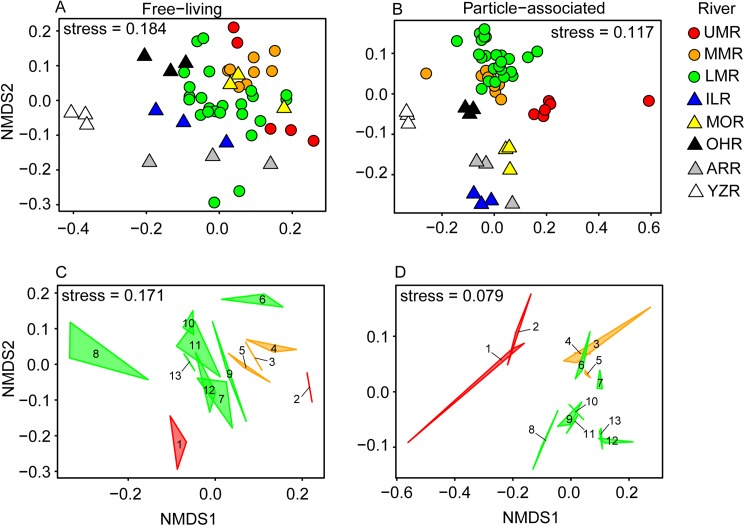
NMDS ordinations of bacterioplankton communities sampled from the Mississippi River system. Ordinations show beta diversity patterns based on Bray-Curtis dissimilarities for (A) free-living and (B) particle-associated communities sampled from the Upper Mississippi River (UMR), Middle Mississippi River (MMR), Lower Mississippi River (LMR), Illinois River (ILR), Missouri River (MOR), Ohio River (OHR), Arkansas River (ARR), and Yazoo River (YZR). Beta diversity patterns for (C) free-living and (D) particle-associated communities from the Mississippi alone are also shown. Images connected at their corners (or line ends) are replicate samples (n = 2–3) from sites on the UMR (red), MMR (orange), and LMR (green). Sites on the Mississippi River are referred to by number, from 1 to 13, from north to south (Table 1). Stress for ordinations are presented in each panel. Ordinations were statistically supported by permutational multivariate analysis of variation (*adonis*; *P* < 0.001 for all).

Beta diversity, or differences in community composition between sites, was assayed by analyzing the relative abundances of OTUs recovered from each site using the Bray-Curtis dissimilarity index, as visualized by non-metric multidimensional scaling (NMDS) ordinations (Fig 5), and comparisons between adjacent sites on the Mississippi River (Table 4). These analyses reveal a number of interesting patterns in spatial relationships of microbiome relatedness across this river system. First, the microbiome of each tributary was distinct for both free-living (Fig 5A) and particle-associated (Fig 5B) bacterial OTUs. Second, both free-living (Fig 5A) and particle-associated (Fig 5B) components of each tributary microbiome were distinct from the Mississippi River microbiome immediately upstream of the confluence. Third, below each major tributary to the Mississippi (the Illinois, Missouri, and Ohio Rivers) there was pronounced divergence in composition of the post-tributary combined community from the pre-tributary community (Fig 5C, Fig 5D, Table 4). In contrast, within sections of the river between major tributaries, or where the tributary was relatively small compared to the Mississippi River (Arkansas and Yazoo Rivers), there was less variation in the Mississippi River microbiome. There were several exceptions to this general pattern. For the free-living community, below the Ohio River from site 6 to site 7 (Fig 5C), there was a large decrease in OTU richness (Fig 4A), and consequently a large disjunction in community diversity. For the particle-associated community, the post-Ohio River confluence site (site 6) was similar to the pre-tributary site (site 5) (Fig 5D; Table 4). Additionally, as noted earlier, at the phylum-level the microbiome of samples from the Mississippi River near the city of Memphis (site 8), was distinct from other sites in the river (Fig 2). These differences were also noted at the OTU-level of analysis, with the Memphis site separating from the other seven sites in the LMR, especially for free-living bacteria (Fig 5C). However, with the exception of Memphis, the microbiome from the pre-Memphis site (site 7) to Natchez, Mississippi (site 13), a stretch of 717 rkm without a major tributary, was relatively stable in composition (Fig 5, Table 4).

**Table 4:** Comparison of Bray-Curtis dissimilarity scores between adjacent sample sites on the Mississippi River.

Site comparison^a^	Intermediate tributary^b^	Free-living	Particle-associated
2 v 1	Illinois	0.45 (0.01)	0.49 (0.06)
3 v 2	Missouri	0.39 (0.01)	0.59 (0.03)
4 v 3	-	0.27 (0.02)	0.43 (0.04)
5 v 4	-	0.28 (0.01)	0.33 (0.01)
6 v 5	Ohio	0.39 (0.01)	0.37 (0.01)
7 v 6	-	0.43 (0.01)	0.40 (0.01)
9 v 7	-	0.34 (0.02)	0.45 (0.02)
10 v 9	-	0.37 (0.02)	0.35 (0.01)
11 v 10	Arkansas	0.29 (0.02)	0.30 (0.01)
12 v 11	-	0.33 (0.02)	0.44 (0.02)
13 v 12	Yazoo	0.27 (0.02)	0.37 (0.02)

Bray-Curtis dissimilarity scores are presented as mean (standard error).

For all comparisons, n = 9 except for sites 2 v 1 (n = 6), sites 3 v 2 (n = 4), and sites 4 v 3 (n = 6).

^a^Site 8 at Memphis was not included because of its apparent influence as a local point source.

^b^Intermediate tributaries are indicated where they occur between adjacent sample sites.

## Discussion

From examining the bacterioplankton microbiome along a 1,300 rkm downriver transect of the Mississippi River, and concurrently at the mouths of five tributaries to the Mississippi, we can reach several important conclusions about the biogeography of the microbiome(s) of this large river system, and perhaps large rivers in general.

The microbiome of each section of the Mississippi, and each tributary was distinct in composition. For the five tributaries, this result was reported in a prior study of the microbial biogeography of this system conducted during summer 2012 [14]. Our results, for a study conducted a year later, confirm that the different rivers of this network have distinct summer microbiomes; additionally, we show this is true for the three major sections of the Mississippi River. As suggested by multivariate correlation analyses of these [14], and other large river networks [11, 16, 22, 39, 40] we assume this spatial pattern in community composition results from differential selection among OTUs in response to the particular environmental properties of each system. An alternative hypothesis to explain inter-river microbiome differences is that exchange of inoculum and colonization among rivers is limited by dispersal barriers. However, considering the various ways in which microbiota might be spread among rivers (wind, barge traffic, animal vectors), even if at low frequency, we think it unlikely that such geographic barriers play an important role compared to the influence of environmental selection [41].

Each river microbiome consisted of free-living and particle-associated assemblages and these two communities were distinct in composition, and presumably biogeochemical processes [42]. These observations emphasize that even in a turbulent and well-mixed river spatial heterogeneity in microbial community composition is possible at the micro-scale as well as at the macro-scale of river basins [11, 14, 43].

Neither the free-living or particle-associated assemblages showed downriver differences in the proportions of dominant bacterial phyla along the Mississippi River, even following major tributary confluences, with the exception of an apparent urban point source of anthropogenic contaminants at Memphis, Tennessee. When sampled over only a two-week period, equilibrium in proportional composition at the phylum-level might be expected for such a broad level of taxonomic resolution, even over so long a river distance. However, it is not at all clear that this would be the case across a longer span in time, for example over the much greater range in physiochemical conditions corresponding to seasonal transitions.

In contrast to the relatively unvarying downriver patterns in diversity at the phylum-level, there was a clear downriver pattern in alpha diversity measures at the OTU-level. Particle-associated bacteria steadily increased in downriver OTU richness. This pattern, we hypothesize, could be a result of increasing particle heterogeneity or particle colonization, or both, with downriver flow. The relatively large increase in OTU richness below the Missouri River can be explained by the high concentration of suspended sediments entering the Mississippi at this confluence, as the Missouri carries a higher sediment load than the UMR. Conversely, the abrupt decline in richness below the Ohio River may be because of dilution with relatively particle-free Ohio River water [23, 24]. Community richness of free-living OTUs also appeared to increase with distance downriver but the pattern was not as pronounced. To explain this difference in patterns of downriver richness, a reasonable hypothesis is that habitat heterogeneity in the mixed fluid phase of flow is less than, and does not increase to the same degree as for the suspended particle load.

Beta diversity of the Mississippi River microbiome varied in general agreement with our predictions regarding the spatial pattern in rates of change with downriver transport. Along the Mississippi there was mostly, but not always, gradual variation in microbiome diversity. This pattern of slow change was especially apparent in the 520 rkm stretch south of Memphis, where the bacterioplankton community was relatively stable. In contrast, below confluences with a major tributary (the Illinois, Missouri, and Ohio Rivers) the microbiome usually, but not always, changed substantially and abruptly in diversity. This was apparent below the Illinois and Missouri Rivers (separating the UMR from the MMR) for both fractions of the microbiome, and for the free-living component below the Ohio River (separating the MMR from the LMR). We assume these downriver punctuations in Mississippi microbiome diversity were due to joining of a major tributary river and its passenger microbiome to the Mississippi microbiome. However, for the Illinois River confluence, there is an alternative explanation. Separating sites 1 and 2 there is the Illinois River, but also a large impoundment, the Melvin Price Dam. This creates more lentic conditions at site 1 and lotic conditions below the dam at site 2, so the change between these two site in the Mississippi microbiome (Fig 5, Table 4) may be an impoundment effect, and not only a tributary effect.

One exception to the usual pattern of generally larger changes of microbiome diversity at major river confluences was for particle-associated assemblages in the LMR below the Ohio River confluence compared to the pre-tributary MMR. This exception can be explained by the fact that the Ohio River carries a much less suspended sediment load, and presumably particle-associated microbes, than the MMR [23, 24]. If this explanation is correct, the particle-associated community below the confluence should be dominated by that of the Mississippi, and this is exactly as we observed. It took 3–4 days, or 325 rkm, of transport below this confluence before the particle-associated component resembled subsequent sites on the LMR. In contrast, from before (site 5) to after (site 6) the Ohio River confluence the free-living microbial community changed relatively strongly, continuing to vary strongly for another day after that, before relative equilibrium was reached over the last 520 rkm of the sampling reach (sites 9–13).

Together, these results suggest a scenario for a pattern in downstream biogeographic variation in river microbiome diversity. At a major confluence, there is neutral mixing of two distinct river microbiomes. This can force a relatively abrupt and large change in diversity of the combined microbiome from the pre-confluence microbiomes, probably by a process of mass immigration. The magnitude of the change will depend on the relative contributions of each river to the merger, a function of individual river discharge and microbiome composition. Following convergence, in response to the new environmental conditions of the merged rivers, and in the absence of other major inputs, there is a more gradual process of microbial community succession. A somewhat similar conclusion for causal factors affecting downriver spatial variation in microbial community structure was reached for the Upper Mississippi River [16, 22].

At Memphis, there was a noticeable change in the microbiome even in the absence of a major tributary input. As opposed to everywhere else, this variation was evident at both low (phylum) and high (OTU) levels of resolution. The bacteria we found associated with the change are indicators of wastewater [44, 45], and probably derived from a treatment plant (MC Stiles Plant), that discharges into the Mississippi River north of downtown Memphis. The influence of anthropogenic inputs at this site is confirmed by a recently published finding that in these samples were elevated concentrations of wastewater contaminants including pharmaceuticals and personal care products [46]. Sampling in an area that might be influenced by such a facility was unintentional, but the results are useful for understanding controls on composition of the river microbiome. Although the shift in the microbiome at Memphis was substantial, it was transient and within one day of transport the pre-city river microbiome had become re-established, with relatively little further change with flow over the next four days. These observations suggest that even in a very large river, microbial contaminants in urban wastewater can alter the composition of a native river microbiome, but their survival may be short-lived compared to indigenous taxa, and hence the disturbance only transient in the adapted, or continuously adapting, river microbiome [22].

## Conclusions

Influenced by its major tributary inputs, the Illinois, Missouri, and Ohio Rivers, the three sections of the Mississippi River (the Upper Mississippi, Middle Mississippi, and Lower Mississippi River) are distinguishable from each other in physical and chemical properties. After one day of flow below confluences with these major tributaries there were usually major shifts in diversity of the Mississippi River microbiome compared to pre-tributary diversity. This is in contrast with stretches of the river more distant in distance and time from major confluences, where changes between adjacent sites in diversity tended to be more gradual. We conclude that while flowing downriver, diversity of the Mississippi River microbiome is modified by point source inputs of external microbiomes from major tributaries draining distinct sub-watersheds, complemented by a gradual process of community succession along intervening stretches of more stable environmental conditions.
